# Quantitative and qualitative perceptions of the 2011 residency duty hour restrictions: a multicenter, multispecialty cross-sectional study

**DOI:** 10.1186/s12909-015-0323-4

**Published:** 2015-03-25

**Authors:** William S Tierney, Rachel L Elkin, Craig D Nielsen

**Affiliations:** Cleveland Clinic Education Institute, 9500 Euclid Avenue, NA2-17, Cleveland, OH 44195 USA

**Keywords:** Residency, Duty hours, Medicine, Surgery, Qualitative, Quantitative, Survey, Intern, Work hours

## Abstract

**Background:**

July 2011 saw the implementation of the newest duty hour restrictions, further limiting the working hours of first year residents and necessitating a variety of adaptations on the part of residency programs. The present study sought to characterize the perceived impact of these restrictions on residency program personnel using a multi-specialty and multi-site approach.

**Methods:**

We developed and administered a survey to internal medicine and general surgery residency programs at three academic medical centers within an urban region. The survey combined quantitative and qualitative components to gain a broader understanding of the impact of the newest regulations. Quantitative responses were compared between Internal Medicine and General Surgery programs with Student t-tests. Other comparisons were performed using ANOVA or Kruskal-Wallis testing as appropriate. For all comparisons, the threshold for significance was set at 0.01. Two independent reviewers coded all qualitative data and assigned one or more themes based on content. Descriptive statistics were calculated and the diversity of themes identified. No between-group comparisons were conducted with the qualitative data.

**Results:**

We found significant differences in the overall perceptions of duty hour restrictions across specialty (internal medicine more positive than general surgery) and across position (first year residents more positive than senior residents and faculty). Notably, individuals who trained at osteopathic medical schools reported significantly more negative views of the duty hour restrictions than those who had trained at allopathic or international medical schools, suggesting an influence of undergraduate medical training. The complementary qualitative data offered insights into the perceived strengths and weaknesses of the duty hour restrictions, as well as actionable suggestions that could help to improve residency program function.

**Conclusion:**

This study characterizes responses to the new duty hour restrictions from a variety of perspectives. Our findings show that individual (type of undergraduate medical education, role in graduate medical education) and program-wide (e.g., specialty) factors contribute to participant satisfaction with DHR. This research highlights the value of a mixed methods approach in the study of duty hour restrictions, with our qualitative arm yielding rich data that complemented and expanded upon the insights derived from the quantitative data.

**Electronic supplementary material:**

The online version of this article (doi:10.1186/s12909-015-0323-4) contains supplementary material, which is available to authorized users.

## Background

The Accreditation Council for Graduate Medical Education (ACGME) instituted new duty hour restrictions (DHR) for residency programs in 2011, limiting postgraduate year (PGY) 1 residents to 16 hours each day, with at least 10 hours in between each active shift [1]. These changes expanded upon previous requirements restricting residents to 80 hour weeks with a maximum of 30 hours in a given shift. Both of these DHR have necessitated significant adaptations by residency programs, which have the potential to alter intern, resident, and faculty perceptions of the new training environment, as well as patient care outcomes.

Previous descriptive studies have sought to characterize the impact of these two duty hour reforms on both residents and faculty. In early surveys following the implementation of the 2003 DHR, residents reported reduced fatigue but felt that patient care had been negatively affected; there were mixed opinions with regards to the impact of the DHR on the educational experience [2,3]. However, a recent nationwide survey of Internal Medicine residents revealed more significant concerns about the adequacy of the resident educational experience [4]. Newer studies conducted after the implementation of the 2011 DHR highlighted additional concerns about continuity of care, PGY1 resident training, and patient safety [5-7].

Early pilot studies of the 2011 DHR yielded mixed results. McCoy et al. [8] did not find significant changes in patient care outcome measures; however, residents reported feeling underprepared to care for patients who had been handed off to them by another team. Another study [9] reported similar concerns about continuity of care and resident preparedness to care for patients. Moreover, no improvements in rest or study time were noted despite fewer hours spent in the hospital.

These concerns have persisted following the nationwide implementation of these DHR. Large national survey studies and a randomized clinical trial noted decreased clinical exposure and overall continuity of care, reduced educational opportunities, and reduced intern preparation for more senior roles as key issues [10-13]. Perceptions of resident fatigue and quality of life in these studies were largely mixed, with many reporting no improvements or even decreases in quality of life and mental health [14]. Taken together, these studies suggest that the 2011 DHR may not be fully realizing its proposed improvements to resident education, patient care, and personal/professional balance [10].

Studies of the impact of the DHR on residency program personnel have focused largely on quantitative data [15], assessed through multiple choice questionnaires or extracted from electronic records. Nearly all of those that did incorporate qualitative data were conducted before the 2011 DHR, and collected the opinions of limited populations [16,17].

Our study sought to gain a broader view of the 2011 DHR by collecting both quantitative and qualitative data. We surveyed interns (PGY1), residents (PGY2-5), and faculty in Internal Medicine (IM) and General Surgery (GS) programs at 3 large academic medical centers in a single metropolitan area. This afforded us the opportunity to make comparisons across sites, across specialties, and across positions.

## Methods

All research was conducted in accordance with the Declaration of Helsinki. The research was approved by the Institutional Review Board (IRB) of each participating institution. The research conducted was considered exempt by all three hospitals’ IRBs because it was conducted without collecting identifying information from participants and was entirely survey-based research. As such, it was deemed to pose little or no risk to participants and written informed consent was not required.

### Setting

Cross-sectional study at three large teaching hospitals in one geographic region. Interns, residents, core faculty, and program directors in IM and GS at these three hospitals were surveyed and all responses were included for analysis.

### Instrument development

We developed a novel survey instrument following a view of the literature and individual interviews with residency Program Directors (PDs) and medical education researchers. Quantitative data were collected via sliding bar questions (0–100 scale with 50 representing neutrality) and multiple choice questions. Qualitative short response questions were optional and placed near the end of the survey. The instrument was validated via trial administration to residents and faculty from non-IM or GS specialties, who were asked to evaluate the face validity, content validity, and construct validity of the survey. (Original survey instrument included as Additional file 1).

### Survey administration

The survey was distributed by PDs to all program residents and core faculty via email using REDCap electronic survey software [18]. Data collection occurred between May 18^th^ and June 30^th^, 2012. All data were collected anonymously and no incentive or compensation was offered to participants. Residency program statistics were obtained directly from each program’s PD via email correspondence.

### Analysis of quantitative data

All statistical analyses were completed using the JMP Pro version 9.0.0 statistical package (© SAS Institute, Inc., Cary, NC). Perceptions of the IM and GS programs were compared with Student t-tests. Group comparisons were performed using ANOVA or Kruskal-Wallis testing as appropriate. Directionality of statistically significant differences was determined using Tukey-Kramer pairwise testing or non-parametric pairwise methods as appropriate. To assess whether each numeric response question was significantly different from a “neutral” score of 50, two-tailed t-tests were utilized. Descriptive statistics were calculated to characterize responses to multiple choice questions. Because of the small sample sizes of each subgroup being compared, we used Wilcoxon Rank Sum and Kruskal-Wallis tests to determine absolute differences between undergraduate training groups and non-parametric pairwise comparisons to determine directionality. To correct for multiple comparisons, a significance threshold of 0.01 was set before beginning statistical analysis.

### Analysis of qualitative data

We used content analysis, more specifically conceptual analysis, to analyze our qualitative data. Data from short response questions were read by two independent reviewers and assigned one or more themes based on content. Coding discrepancies were settled via collaborative discussion. When possible, similar concepts were condensed into a single, unifying theme; this process was repeated iteratively until no such condensations were possible. We then calculated descriptive statistics (counts and percentages) for each of the remaining themes. No comparisons of qualitative data between groups were conducted given the smaller respondent pool (these questions were optional) and the diversity of themes identified. The analyses and conclusions reported for the qualitative data thus are reflective of the entire respondent pool for these questions.

## Results and discussion

We received 202 responses before study closure: 34 faculty, 98 PGY1 residents, and 70 senior residents (PGY2+). Our resident response rate was 49% (168/341). 62 (31%) respondents reported affiliation with Hospital A, 76 (38%) with Hospital B, and 45 (22%) with Hospital C; 19 (9%) of respondents did not report a hospital affiliation. Key demographic data for our population are summarized in Table 1.

**Table 1:** **Demographic characteristics of survey respondents**

	PYG1	PYG2+	Faculty
Survey respondents*	70 (35%)	98 (49%)	34 (17%)
Hospital affiliation*			
*Hospital A*	23 (37%)	22 (35%)	17 (27%)
*Hospital B*	74 (32%)	44 (58%)	8 (11%)
*Hospital C*	14 (31%)	6 (13%)	25 (56%)
*No hospital identified*	9 (47%)	7 (37%)	3 (16%)
Specialty*			
*Internal medicine*	65 (37%)	84 (49%)	23 (14%)
*General surgery*	3 (15%)	8 (40%)	9 (45%)
*Other*	4 (33%)	6 (50%)	2 (17%)

*n(%).

### Quantitative data

*All respondents* (Table 2): Mean perceptions of the effect of DHR on continuity of care, ability to learn new procedures, ability to practice procedures, and ability to teach were all statistically worse than neutral (all p<0.0001). Overall perceptions of the DHR, the impact of the DHR on ability to learn new medical material, and the impact of the DHR on ability to review previously learned material were not statistically different from neutral (p=0.1192, p=0.8051, and p=0.7748, respectively). None of the quantitative domains assessed showed a mean better-than-neutral response that reached statistical significance.

**Table 2:** **Quantitative perceptions of DHR - all respondents**

Question	Mean response	t-test for neutral
General perception	46.8	p=0.1192
Continuity of care	36.9	p<0.0001*
Learning new procedures	41.8	p<0.0001*
Practicing known procedures	44.8	p<0.0001*
Learning new medical knowledge	48.8	p=0.8051
Ability to review medical knowledge	51.1	p=0.7748
Ability to teach	40.7	p<0.0001*

*statistically worse than neutral.

Participants were asked additional questions about some of the domains that the new regulations specifically were designed to improve. Both PGY1 and PGY2+ residents reported devoting a median of 5 hours (IQR: 3–10) to independent study per week. The average amount of sleep reported by participants was 6.4 ± 1.2 hours per night (range 1–9; PGY1: 6.5 ± 0.7, PGY2+: 6.4 ± 1.5, faculty: 6.3 ± 1.0). Only 65% (PGY1: 61%, PGY2+: 67%, faculty: 67%) of participants were satisfied with this amount of sleep, however.

When asked if the new DHR had affected the likelihood of their pursuing a career in academic medicine, most (70% of PGY1 respondents, 87% of PGY2+ respondents) reported that the DHR had no impact on the likelihood of them pursuing an academic career. 20% of PGY2+ residents and 8.7% of PGY1 residents reported that the new DHR had reduced the likelihood of them pursuing an academic career, while 10% and 4.3%, respectively, reported an increased likelihood.

*Cross-site comparisons* (Table 3): There were no significant differences across sites (Hospitals A, B, and C) for any of the quantitative questions asked, except in the ability of residency program participants to learn new procedures (p=0.004).

**Table 3:** **Quantitative perceptions of DHR across hospitals**

Question	Means Hospital A/B/C	ANOVA
General perception	43.0/44.5/53.2	P=0.3052
Continuity of care	37.6/32.3/37.1	P=0.2775
Learning new procedures	38.8/39.4/46.5	P=0.004*^‡^
Practicing known procedures	40.8/46.0/47.0	P=0.0499
Learning new medical knowledge	47.7/46.1/51.4	P=0.5151
Ability to review medical knowledge	52.4/45.0/54.4	P=0.0888
Ability to teach	38.1/39.0/43.4	P=0.3312

*statistically significant finding, ^‡^on pairwise comparison, Hosp. C statistically different from Hospital A/B/C.

*Cross-position comparisons* (Table 4): PGY1 residents rated their overall perception of the DHR more positively than both PGY2+ residents and faculty (p<0.0001). Similarly, PGY1 residents rated the impact of the DHR on their ability to teach more favorably than PGY2+ residents and faculty (p<0.0001). PGY1 residents rated the impact of the DHR on continuity of care more favorably than faculty (p=0.0038), but were not statistically different from PGY2+ residents.

**Table 4:** **Quantitative perceptions of DHR across positions**

Question	Means PGY1/PGY2+/Faculty	ANOVA
General perception	60.9/41.2/34.2	p<0.0001*^‡^
Continuity of care	43.7/35.1/28.0	p=0.0038*^†^
Learning new procedures	41.7/40.1/46.9	p=0.1704
Practicing known procedures	45.3/43.2/48.2	p=0.3435
Learning new medical knowledge	53.7/46.6/45.6	p=0.0508
Ability to review medical knowledge	54.1/50.1/47.5	p=0.2350
Ability to teach	50.4/38.8/31.3	p<0.0001*^‡^

*statistically significant finding, ^‡^on pairwise comparison, PGY1 group statistically more positive than PGY2+ or Faculty (p<0.01), ^†^on pairwise comparison, PGY1 group statistically more positive than faculty (p<0.01) but not PGY2+ (p>0.01).

*Cross-specialty comparisons* (Table 5): At a single site (Hospital B), we compared 20 GS respondents to 29 IM respondents. We observed a significant difference between GS and IM programs in their overall perceptions of the DHR (p=0.0057). No other significant differences were identified across the two groups.

**Table 5:** **Quantitative perceptions of DHR between specialties (one hospital)**

Question	Means GS/IM	t-test
General perception	31.0/51.9	p=0.0057*
Continuity of care	25/37.2	p=0.0206
Learning new procedures	39.3/38.3	p=0.7848
Practicing known procedures	42.2/46.5	p=0.2533
Learning new medical knowledge	41.2/46.5	p=0.2777
Ability to review medical knowledge	42.3/43.0	p=0.8864
Ability to teach	35.7/40.2	p=0.3582

*statistically significant finding.

*Impact of undergraduate medical training program*: We compared residents based on type of undergraduate medical education (US/Canadian allopathic, US/Canadian osteopathic, international medical school). While the core curricular basic science and clinical content is largely similar amongst the three, osteopathic schools advocate a more holistic approach to patient care, place more emphasis on preventive medicine and primary care, and provide special training in osteopathic manipulative medicine (a core set of techniques typically applied in the treatment of musculoskeletal disorders).

With regards to overall perceptions of the DHR, osteopathic graduates were less satisfied than either allopathic or international graduates (means 27, 46, 52 respectively; Wilcoxon Rank Sum p=0.0051). Similarly, osteopathic graduates felt that the DHR more negatively impacted continuity of care and their ability to teach (Wilcoxon Rank Sum p=0.0075 and p=0.0002 respectively). No statistically significant differences were detected between allopathic and international graduate respondents.

### Qualitative data

127/202 (63%) respondents described weaknesses of DHR, while 121/202 (60%) provided strengths. 68/202 (34%) offered specific suggestions to improve the function of residency programs. Responses describing DHR weaknesses tended to be longer than those describing strengths (mean of 131 versus 50 characters/comment).

The weaknesses most commonly identified by survey participants were reduced continuity of care (25.7% of respondents); increased handoff frequency (16.8%); reduced intern preparedness for more senior positions (9.4%); that the new DHR promoted the adoption of a “shift mentality” (8.9%); and that the new DHR brought with it a disproportionate increase in the senior resident workload (8.4%).

Key strengths identified by respondents included better rested residents (22.8%); better personal/professional balance (14.4%); increased independent study time (8.9%); and improved patient safety (3.5%). Some also felt that the newest DHR were more reasonable as compared to previous iterations (3.5%).

The most common suggestions mentioned by participants focused on removing the 16 hour daily limit imposed on PGY1 residents (4.5%) and expanding the use of “night float” services (4.5%). Other respondents spoke to the need to revise and expand the formal educational curriculum within the context of the new DHR (4.0%); to identify ways to maximize the efficiency of resident time spent at the hospital (e.g., reducing resident administrative burden, improving handoff efficiency; 3.5%); and to increase the amount of contiguous time off that residents receive (3.0%).

A complete listing of all themes collected from the short response questions may be found in Additional file 2: Table S1, Additional file 3: Table S2 and Additional file 4: Table S3.

## Conclusions

Perceptions of the impact of the DHR vary widely. The present study suggests that both individual (type of undergraduate medical education, role in graduate medical education) and program-wide (e.g., specialty) factors contribute to participant satisfaction with the newest DHR. We found that PGY1 residents tend to view most aspects of the DHR more favorably than more senior residents or faculty. Individuals in IM also tended to feel more positively about the DHR compared with their GS counterparts. Finally, we identified a potential role for undergraduate medical education in shaping the perceptions of the new DHR, with osteopathic graduates rating the DHR more negatively in several domains relative to their allopathic and international graduate colleagues.

The qualitative results of this research illustrate the most commonly perceived strengths and weaknesses of the new DHR and present useful suggestions to improve residency program function. Our respondents felt that while the new DHR have brought improvements in time for resident rest, patient safety, and personal/professional balance, there remains room for improving the continuity of care, intern preparation for more senior positions, and balancing senior resident workload. Many of the suggestions sought to optimize the function of residency programs within the context of the DHR, but a substantial portion called for a fundamental restructuring of the 2011 DHR to be able to improve program function most effectively.

Senior residents and faculty – the two groups able to compare the old and new duty hour paradigms – rated many aspects of the new DHR more negatively than PGY1 residents. This suggests that many individuals who have experienced both paradigms are less satisfied with the newest iteration of the DHR, at least after its first year of implementation. Similarly, individuals in the GS residency program reported more dissatisfaction with the DHR than those in IM in the quantitative arm of our study. However, the only significant difference across specialties was in the overall perceptions of the duty hours; no significant differences were found with regards to continuity of care or ability to learn, for example. This may be due to the relatively small subgroups being assessed or it may reflect a difference in participant perceptions of how they are able to perform within their residency programs as opposed to their perceptions of DHR as a concept.

The discrepancies in DHR perceptions based upon type of undergraduate medical education were an unexpected and novel finding. On the whole, the ACGME and American Osteopathic Association DHR are fairly similar [1,19], suggesting that differences in expectations of working hours do not underlie these differences. These findings may reflect differences in the culture of osteopathic and allopathic undergraduate training programs, or perhaps may be a form of selection bias reflecting the views of those osteopathic trainees who elected to enter an allopathic residency program as opposed to the perceptions of the osteopathic community as a whole. Additional research will be required to understand the implications of these results more fully. We found no reports of the impact of undergraduate medical education on perceptions of resident duty hours. Our findings suggest that this variable may be an important influence on views of the DHR, and we recommend that future studies of the DHR consider collecting this data.

One of the strengths of this study was the inclusion of qualitative, free response questions to complement our multiple choice questions. Previous research on the 2011 duty hours has relied primarily on quantitative survey methodology. Our qualitative data therefore help to add another dimension to the duty hours literature. The qualitative questions revealed strengths and weaknesses of the 2011 DHR that would not have been identified using quantitative methods alone. Inclusion of qualitative questions thus may be a simple way to increase the scope of possible survey responses. Additionally, our qualitative survey item asking for suggestions yielded many specific and actionable ideas. While each residency program is different, use of similar questions in an internal survey format could be a mechanism for programs to drive positive change from within.

Some limitations to the present study should be acknowledged. First, we only received responses from one GS program, limiting the power of the cross-specialty comparisons. Second, there is the potential for response bias, in both our quantitative and qualitative arms. It is of note, though, that many of the trends and themes identified in the present survey are consistent with those reported in national surveys of IM and GS residents and program directors [10,13].

From length and content analyses, study participants tended to have stronger and more plentiful comments on the weaknesses of the DHR than the strengths. These findings suggest that residency program participants feel more negatively about duty hours, or perhaps that the culture within residency programs is to emphasize the negative aspects of DHR. On the other hand, in the quantitative arm of the study, the average overall perception of the DHR was not significantly worse than neutral. Therefore, a discrepancy exists between the qualitative and quantitative feedback received. This may represent a selection bias with regards to who elected to respond to the qualitative questions or it may indicate a disconnect between quantitative and qualitative perceptions of the DHR. Whether individuals simply find more specific critiques than positive comments when considering the DHR or people with critiques are more likely to complete qualitative questions is not clear from this research. An alternative explanation is that this discrepancy between our qualitative and quantitative data originates because open-ended qualitative questions allowed respondents to comment on some negative aspect of the DHR which was not represented in our quantitative survey items. However, after careful review of all qualitative data we were unable to identify such a factor and this is an unlikely explanation.

### Ethical statement

See Methods section.

## Additional files

Additional file 1:
**Original survey instrument used to collect data in this study.**
Additional file 2: Table S1. Complete Weaknesses. Additional file 3: Table S2. Complete Strengths. Additional file 4: Table S3. Complete Suggestions.

## Abbreviations

ACGME: Accreditation Council for Graduate Medical Education
DHR: Duty hour restriction
PGY: Post-graduate year (for example PGY1 for post-graduate year 1)
IM: Internal medicine
GS: General surgery
PD: Program director
US: United States of America
IRB: Institutional Review Board
