# Supplementary material for: Quantitative and qualitative perceptions of the 2011 residency duty hour restrictions: a multicenter, multispecialty cross-sectional study
Source: BMC Med Educ. 2015 Mar 25;15:57. doi: 10.1186/s12909-015-0323-4 (PMC4403846; doi:10.1186/s12909-015-0323-4)
Supplement: Additional file 1: — Original survey instrument used to collect data in this study. [file 12909_2015_323_MOESM1_ESM.pdf]

Survey

This survey takes 3-5 minutes to complete.

If for any of the "slider" questions you would like to select the neutral/no impact/no difference option, please click the bar once, and set it to a value of 50.

Please state your current position.

- ☐ Intern
- ☐ Resident
- ☐ Chief Resident
- ☐ Fellow
- ☐ Attending Physician
- ☐ Associate Program Director
- ☐ Program Director
- ☐ Other

Are you aware of the new duty hour requirements implemented in July 2011?

- ☐ No
- ☐ Yes

How do you feel about the new duty hour requirements?

Very Negatively | Neutral | Very Positively

(Place a mark on the scale above)

How do you feel that the new duty hours affect continuity of patient care in your field?

Strong negative impact on continuity of care | No impact | Strong positive impact on continuity of care

(Place a mark on the scale above)

How have the new duty hours impacted your ability to learn new surgical or procedural techniques?

Much harder for me to learn new surgical or procedural techniques | No difference | Much easier for me to learn new surgical or procedural techniques

(Place a mark on the scale above)

How have the new duty hours impacted your ability to practice previously learned surgical or procedural techniques?

Much harder for me to practice previously learned surgical or procedural techniques | No difference | Much easier for me to practice previously learned surgical or procedural techniques

(Place a mark on the scale above)

How have the new duty hours impacted your ability to acquire new medical knowledge?

Much harder for me to acquire new medical knowledge | No difference | Much easier for me to acquire new medical knowledge

(Place a mark on the scale above)

How have the new duty hours impacted your ability to review previously acquired medical knowledge?

Much harder for me to review previously acquired medical knowledge | No difference | Much easier for me to review previously acquired medical knowledge

(Place a mark on the scale above)

In your current position, do you teach residents or medical students?

- ☐ No
- ☐ Yes

How have the new duty hours impacted your ability to teach?

Negatively impact my ability to teach — No impact — Positively impact my ability to teach

(Place a mark on the scale above)

The new duty hours have affected my teaching in the following ways: (Check all that apply)

- ☐ Decreased the amount of time I have to teach
- ☐ Increased the amount of time I have to teach
- ☐ Less patient cases available to use for teaching
- ☐ More patient cases available to use for teaching
- ☐ Less time for non-GME, non-patient care-related activities
- ☐ More time for non-GME, non-patient care-related activities

The time I spend teaching the following groups has decreased (Check all that apply)

- ☐ Medical Students
- ☐ Interns
- ☐ Residents
- ☐ Allied Health Students

The time I spend teaching the following groups has increased (Check all that apply)

- ☐ Medical Students
- ☐ Interns
- ☐ Residents
- ☐ Allied Health Students

During the past month, how many hours of sleep do you get on average per night? (Round to the nearest hour)

____

Do you feel that this amount of sleep is adequate for your needs?

- ☐ No
- ☐ Yes

Do you feel that your responsibilities/workload are:

- ☐ Insufficient given the amount of time I spend in the hospital
- ☐ Adequate given the amount of time I spend in the hospital
- ☐ Excessive given the amount of time I spend in the hospital

On an average workday, how many hours do you spend at the hospital?

____

Do you feel that the new duty hour requirements have influenced the teaching style of the residents on your service?

- ☐ No
- ☐ Yes
- ☐ Not applicable

If you answered "yes" above, please explain. (Check all that apply)

- ☐ The residents have developed a more efficient teaching style.
- ☐ The residents have developed a more learner-centered teaching style
- ☐ The residents have less time to devote to teaching, but they have not changed their teaching style.
- ☐ The residents seem more harried (anxious, irritable, or stressed about their responsibilities) when teaching.
- ☐ The residents have spent less time performing bedside teaching.
- ☐ Other

If you chose "other" above, please explain.

____

Do you feel that the new duty hour requirements have influenced the teaching style of the attendings on your service?

- ☐ No
- ☐ Yes
- ☐ Not applicable

If you answered "yes" above, please explain. (Check all that apply)

- ☐ The attendings have developed a more efficient teaching style.
- ☐ The attendings have developed a more learner-centered teaching style
- ☐ The attendings have less time to devote to teaching, but they have not changed their teaching style.
- ☐ The attendings seem more harried (anxious, irritable, or stressed about their responsibilities) when teaching.
- ☐ The attendings have spent less time performing bedside teaching.
- ☐ Other

If you chose "other" above, please explain. ______________________________

Do you feel that the new duty hours have influenced your likelihood to pursue a career in academic medicine?

- ☐ The new duty hours make me less likely to pursue a career in academic medicine
- ☐ The new duty hours have made no impact on my future plans
- ☐ The new duty hours make me more likely to pursue a career in academic medicine

When you are not at the hospital, how many hours per week on average, do you spend studying material directly relevant to your field? ______________________________

What do you feel are the strengths of the new duty hour system? ______________________________

What do you feel are the weaknesses of the new duty hour system? ______________________________

What suggestions, if any, do you have for your training program as it adapts to the new duty hour requirements? ______________________________

Overall, do you feel that compliance with the new duty hour requirements will provide you with adequate preparation for future fellowship and staff positions?

- ☐ No
- ☐ Yes

What hospital campus have you been working in for the past month?

- ☐ Cleveland Clinic Main Campus
- ☐ Cleveland Clinic Satellite Campus
- ☐ Louis Stokes Cleveland VA Medical Center
- ☐ MetroHealth Medical Center
- ☐ MetroHealth Satellite Campus
- ☐ University Hospitals Case Medical Center
- ☐ University Hospitals Satellite Campus
- ☐ Other

Please state your specialty. If you are a resident, please indicate the program you are currently completing, not an intended subspecialty.

- ☐ Allergy and Immunology
- ☐ Anesthesiology
- ☐ Dermatology
- ☐ Emergency Medicine
- ☐ Family Medicine
- ☐ General Surgery (including subspecialties)
- ☐ Hospice and Palliative Medicine
- ☐ Internal Medicine (including subspecialties)
- ☐ Medical Genetics
- ☐ Neurological Surgery
- ☐ Neurology
- ☐ Nuclear Medicine
- ☐ Ophthalmology
- ☐ Orthopaedic Surgery
- ☐ Otolaryngology
- ☐ Pathology (including subspecialties)
- ☐ Pediatrics
- ☐ Physical Medicine and Rehabilitation
- ☐ Plastic Surgery
- ☐ Preventative Medicine
- ☐ Psychiatry
- ☐ Radiation Oncology
- ☐ Radiology - Diagnostic
- ☐ Sleep Medicine
- ☐ Thoracic Surgery
- ☐ Urology
- ☐ Other

Residents and Fellows: Please state the specialty you intend to enter after you complete all of your training (residency, fellowship(s), etc.).

- ☐ Advanced Heart Failure and Transplant Cardiology
- ☐ Adolescent Medicine
- ☐ Aerospace Medicine
- ☐ Allergy and Immunology
- ☐ Anesthesiology
- ☐ Cardiovascular Disease
- ☐ Clinical Cardiac Electrophysiology
- ☐ Colon and Rectal Surgery
- ☐ Critical Care Medicine
- ☐ Dermatology
- ☐ Emergency Medicine
- ☐ Endocrinology, Diabetes and Metabolism
- ☐ Family Medicine
- ☐ Gastroenterology
- ☐ General Surgery
- ☐ Geriatric Medicine
- ☐ Hematology
- ☐ Hospice and Palliative Medicine
- ☐ Infectious Disease
- ☐ Internal Medicine
- ☐ Interventional Cardiology
- ☐ Medical Genetics
- ☐ Medical Oncology
- ☐ Nephrology
- ☐ Neurological Surgery
- ☐ Neurology
- ☐ Nuclear Medicine
- ☐ Obstetrics and Gynecology
- ☐ Occupational Medicine
- ☐ Ophthalmology
- ☐ Orthopaedic Surgery
- ☐ Otolaryngology
- ☐ Pathology
- ☐ Pediatrics
- ☐ Physical Medicine and Rehabilitation
- ☐ Plastic Surgery
- ☐ Psychiatry
- ☐ Public Health and General Preventive Medicine
- ☐ Pulmonary Disease
- ☐ Radiology
- ☐ Rheumatology
- ☐ Sleep Medicine
- ☐ Sports Medicine
- ☐ Thoracic and Cardiac Surgery
- ☐ Transplant Hepatology
- ☐ Vascular Surgery
- ☐ Urology

Please indicate the year in which you graduated medical school. ______________________________

If you are a resident/fellow, what is your PGY? (If you are PGY1, enter "1" below). ______________________________

If you are an attending, in what year did you begin your involvement in GME as an attending physician? ______________________________

Where did you complete your undergraduate medical training?

- ☐ Allopathic US Medical School
- ☐ Osteopathic US Medical School
- ☐ International Medical School

Please estimate the number of students in your graduating medical school class. ______________________________
