# Supplementary material for: Quantitative and qualitative perceptions of the 2011 residency duty hour restrictions: a multicenter, multispecialty cross-sectional study
Source: BMC Med Educ. 2015 Mar 25;15:57. doi: 10.1186/s12909-015-0323-4 (PMC4403846; doi:10.1186/s12909-015-0323-4)
Supplement: Additional file 2: Table S1. — Complete Weaknesses. [file 12909_2015_323_MOESM2_ESM.pdf]

Supplemental Table 1 - Complete Weaknesses	Count	%Respondents
Reduced continuity of patient care	52	17.87%
More handoffs	34	11.68%
Residents less prepared after intern year (decreased preparation)	19	6.53%
Shift mentality	18	6.19%
Senior Resident Workload	17	5.84%
Reduced Procedures	16	5.50%
Less Teaching	16	5.50%
Decreased Patient Exposure	14	4.81%
Less rest	14	4.81%
Decreased overnight experience (quantity and quality)	13	4.47%
Shift work	12	4.12%
Decreased admission experience	11	3.78%
Decreased learning opportunities	11	3.78%
Decreased quality of patient care	7	2.41%
Reduced Autonomy	6	2.06%
Increased workload	6	2.06%
Reduced time in hospital/reduced time to work on cases	5	1.72%
Decreased Resident/Intern Overlap	4	1.37%
Decreased educational continuity	4	1.37%
Violation of duty hours	4	1.37%
Less independent study time	3	1.03%
More busy work	2	0.69%
Decreased teamwork	2	0.69%
Confusing for patients	1	0.34%
