# Supplementary material for: Quantitative and qualitative perceptions of the 2011 residency duty hour restrictions: a multicenter, multispecialty cross-sectional study
Source: BMC Med Educ. 2015 Mar 25;15:57. doi: 10.1186/s12909-015-0323-4 (PMC4403846; doi:10.1186/s12909-015-0323-4)
Supplement: Additional file 3: Table S2. — Complete Strengths. [file 12909_2015_323_MOESM3_ESM.pdf]

Supplemental Table 2 - Complete Strengths	Count	%Respondents
Better rested	46	22.77%
Personal/professional balance (increased personal time)	29	14.36%
Increased time for self-study	18	8.91%
Better patient safety	7	3.47%
More reasonable hours	7	3.47%
Increased personal efficiency	4	1.98%
Better patient care	4	1.98%
Better handoffs	4	1.98%
Night float - positive	3	1.49%
Better learning	3	1.49%
Well-enforced	3	1.49%
Less resident/intern abuse	2	0.99%
Continuity of care preserved	2	0.99%
Improved curricular efficiency	2	0.99%
Better teaching	1	0.50%
Residents are safer	1	0.50%
More patient exposure	1	0.50%
