# Supplementary material for: Quantitative and qualitative perceptions of the 2011 residency duty hour restrictions: a multicenter, multispecialty cross-sectional study
Source: BMC Med Educ. 2015 Mar 25;15:57. doi: 10.1186/s12909-015-0323-4 (PMC4403846; doi:10.1186/s12909-015-0323-4)
Supplement: Additional file 4: Table S3. — Complete Suggestions. [file 12909_2015_323_MOESM4_ESM.pdf]

Supplemental Table 3 - Complete Suggestions	Count	%Respondents
Increase the use of a night float system	9	4.46%
Remove 16 hour daily limit	9	4.46%
More formal educational activities	8	3.96%
Improve Continuity of Care	7	3.47%
Refine current system for more effective/efficient operation/team dynamic	7	3.47%
More contiguous time off	6	2.97%
Increased Admission Experience	4	1.98%
Increased Procedure Experience	4	1.98%
Standardize Signouts and Handoffs	4	1.98%
Take measures to ensure that duties can be adequately fulfilled within current limits	4	1.98%
Better coordination of teams (balance, schedules, etc.)	4	1.98%
Increase number of mid-level providers	3	1.49%
Increase intern supervision	2	0.99%
Increased time spent in hospital	2	0.99%
Senior residents should not be given more work because of PGY1 DHR	1	0.50%
Implement non-teaching clinical services	1	0.50%
No more reduction in DHs	1	0.50%
Cover fewer services per year/educational continuity	1	0.50%
Get rid of night float - need more intern supervision at night	1	0.50%
No continuity clinic while on inpatient service	1	0.50%
Formal inclusion of EBM in the curriculum	1	0.50%
